# Real-life data of patients with chronic myeloid leukemia in Amazonas

**DOI:** 10.1186/s12885-026-15843-4

**Published:** 2026-03-25

**Authors:** Rosângela Santos Abreu, Kasthyhellen Souto Maior, Iandara Maira Lopes Souza, Jhemerson Fernandes Paes, Bruno Eduardo Feitosa Nascimento, Rafaella Oliveira Santos, Nadja Pinto Garcia Romero, Júlia Pereira Martins, Allyson Guimarães Costa, Adriana Malheiro, Olindo Assis Martins-Filho, Andréa Monteiro Tarragô, Leny Nascimento Motta Passos

**Affiliations:** 1 Programa de Pós-Graduação em Ciências Aplicadas à Hematologia, Universidade do Estado do Amazonas (UEA), Manaus, Brazil; 2 Diretoria de Ensino e Pesquisa, Fundação Hospitalar de Hematologia e Hemoterapia do Amazonas (HEMOAM), Av. Constantino Nery 4397, Chapada, Manaus, AM 69050-001 Brazil; 3 Centro Universitário Nilton Lins, Manaus, Brasil; 4 Centro Universitário FAMETRO, Manaus, Brazil; 5 Programa de Pós-Graduação em Imunologia Básica e Aplicada, Instituto de Ciências Biológicas, Universidade Federal do Amazonas (UFAM), Manaus, Brazil; 6 Programa de Pós-Graduação em Biotecnologia, Instituto de Ciências Biológicas, Universidade Federal do Amazonas (UFAM), Manaus, Brazil; 7 Instituto René Rachou, Fundação Oswaldo Cruz (FIOCRUZ- MINAS), Belo Horizonte, Brazil

**Keywords:** Chronic myeloid leukemia, Sokal score, Molecular response, *BCR*::*ABL1*, Tyrosine kinase inhibitors

## Abstract

**Background:**

Chronic myeloid leukemia (CML) is a myeloproliferative neoplasm caused by the t(9;22)(q34;q11.2) translocation, known as the Philadelphia chromosome (Ph+), which generates the *BCR::ABL1* fusion oncogene central to CML pathogenesis. The disease accounts for approximately 15% of adult leukemias, predominantly affects males, and has a median age at diagnosis of around 57 years. CML typically presents in the chronic phase and may progress to advanced disease phases, with prognostic risk stratification guiding treatment decisions. Tyrosine kinase inhibitors (TKIs) are the cornerstone of therapy, aiming to achieve durable molecular disease control and, in selected patients, sustained deep molecular response and treatment-free remission. This study evaluated the demographic, clinical, and molecular response profiles of patients with Ph + CML treated with first- and second-line TKIs at the Fundação Hospitalar de Hematologia e Hemoterapia do Amazonas (HEMOAM).

**Methods:**

This retrospective, longitudinal study analyzed medical records of 176 patients diagnosed with Ph + CML between 2011 and 2020. Demographic characteristics, clinical data, and laboratory findings were extracted from medical records. Longitudinal molecular response was assessed in patients with available *BCR::ABL1* monitoring.

**Results:**

Among the 176 patients identified, 122 were included in the analysis of treatment outcomes. The mean age at diagnosis was 49.6 years, with a slight male predominance. Most patients were diagnosed in the chronic phase, and high-risk Sokal score was the most frequent prognostic category among evaluable cases. The e14a2 (b3a2) transcript was the most prevalent molecular subtype. Clinically meaningful molecular responses were observed in both first- and second-line treatment settings, although response assessment was influenced by real-world constraints, including incomplete molecular monitoring and heterogeneous follow-up.

**Conclusions:**

These findings provide real-world insights into the epidemiological, clinical, and molecular response characteristics of Ph + CML patients treated in the Amazon region. The study highlights the challenges of implementing guideline-based molecular monitoring in geographically constrained settings and supports the need for adapted strategies to optimize long-term CML management in routine clinical practice.

## Background

Chronic myeloid leukemia (CML) is a malignant hematopoietic stem cell disorder caused by the reciprocal translocation t(9;22)(q34;q11.2), known as the Philadelphia chromosome (Ph), which generates the *BCR::ABL1* fusion gene and drives uncontrolled proliferation of the leukemic clone [1–3]. The disease has an incidence of approximately 1–2 cases per 100,000 individuals per year, accounting for nearly 15% of adult leukemias and less than 3% of pediatric cases. CML predominantly affects males, with a reported male-to-female ratio ranging from 1.2:1 to 1.7:1, and a median age at diagnosis of approximately 57 [4–7].

The breakpoint in the ABL1 gene typically occurs between exons 1a and a2 and is fused to the BCR gene on chromosome 22. The most frequent breakpoint region is the major breakpoint cluster region (M-BCR), resulting in b2a2 (e13a2) and b3a2 (e14a2) fusion transcripts, which encode the p210 *BCR::ABL1* protein in approximately 95% of cases [8–11]. Clinically, CML is classically described as a triphasic disease comprising chronic, accelerated, and blast phases, with most patients diagnosed during the chronic phase. Diagnosis is established through clinical evaluation, laboratory findings, cytogenetic and/or molecular confirmation of *BCR::ABL1*, and prognostic risk stratification [12, 13].

Molecular monitoring using quantitative reverse transcription polymerase chain reaction (RT-qPCR) enables sensitive detection of residual disease and longitudinal assessment of treatment response to tyrosine kinase inhibitors (TKIs) [14, 15]. Current treatment strategies are centered on TKIs, including imatinib as first-line therapy and dasatinib, nilotinib, or bosutinib as alternative first- or second-line options, with ponatinib or asciminib reserved for later treatment lines [14, 16, 17]. In Brazil, CML management within the public healthcare system follows guidelines established by the Unified Health System (SUS) and the National Commission for the Incorporation of Technologies (CONITEC), largely based on European LeukemiaNet recommendations [18].

Over the past two decades, the introduction of TKIs has transformed the natural history of CML, enabling most patients to achieve durable molecular responses and long-term survival approaching that of the general population. In selected patients, sustained deep molecular responses may allow treatment discontinuation and treatment-free remission, often referred to as a “functional cure” [19, 20]. In this context, real-world data are essential to understand how these therapeutic advances translate into routine clinical practice, particularly in regions facing structural and geographic barriers to healthcare access.

The Fundação Hospitalar de Hematologia e Hemoterapia do Amazonas (HEMOAM) is the main referral center for hematologic malignancies in the state of Amazonas and is responsible for the care of nearly all patients with CML in the region. This study aimed to characterize the demographic, clinical, and molecular response profiles of patients with Ph-positive CML treated at HEMOAM, providing real-world insights into disease management in a geographically and logistically challenging setting.

## Methods

### Ethics statement

The research project was submitted to and approved by the Ethics Committee of the Fundação Hospitalar de Hematologia e Hemoterapia do Amazonas (HEMOAM) under approval number 4.632.515. All participants provided written informed consent prior to inclusion in the study, in accordance with Resolution No. 466/2012 of the Brazilian National Health Council and ethical principles of the Declaration of Helsinki for research involving human subjects.

### Study design

This was an observational, retrospective, longitudinal, and descriptive study conducted at HEMOAM, the main referral center for hematologic diseases in the state of Amazonas. The target population included patients diagnosed with Philadelphia chromosome–positive (Ph+) chronic myeloid leukemia (CML) at HEMOAM who received treatment with tyrosine kinase inhibitors (TKIs) between January 1, 2011 and December 31, 2020.

A total of 176 patients with Ph + CML were identified during the study period. Of these, 122 patients diagnosed in the chronic phase were included in the longitudinal clinical and molecular response analyses. Fifty-four patients were excluded due to absence of molecular monitoring data at any timepoint or loss to follow-up.

All patients were initially treated with imatinib as first-line therapy, in accordance with national treatment guidelines during the study period. Patients who developed intolerance, confirmed treatment failure, or persistent suboptimal response were switched to second-generation TKIs (dasatinib or nilotinib), based on clinical characteristics, comorbidities, and drug availability within the public healthcare system.

### Eligibility criteria and definition of chronic phase

Therapeutic monitoring analyses were restricted to adult patients (≥ 18 years) of either sex, diagnosed with Ph + CML in the chronic phase, and who underwent at least one molecular assessment of *BCR::ABL1*during follow-up.

Chronic phase was defined according to standard clinical and laboratory criteria, consistent with World Health Organization and European LeukemiaNet recommendations, including the absence of features of accelerated phase or blast crisis at diagnosis, such as marked blast excess, severe thrombocytopenia or thrombocytosis, and extramedullary blast proliferation.

Patients were excluded from the therapeutic monitoring analyses if they were younger than 18 years, diagnosed in accelerated phase or blast crisis, had concomitant hepatitis B, hepatitis C, or HIV infection, advanced hepatic disease, advanced-stage non-hematologic malignancies, documented lack of regular treatment adherence, or absence of molecular monitoring data.

### Clinical and laboratory data collection

Data were obtained through a comprehensive review of physical and electronic medical records, accessed via the Patient Records Service (SPP) and the I-doctor system, an integrated hospital management platform. Information was systematically recorded using standardized data collection forms.

Collected variables included demographic characteristics (year of diagnosis, age, sex, and municipality of origin), clinical data (disease phase at diagnosis, Sokal risk score when available, date of treatment initiation, and type of TKI therapy), and laboratory parameters. Laboratory data included complete blood count at diagnosis and qualitative and/or quantitative *BCR::ABL1* molecular test results obtained during follow-up.

### Molecular response assessment

Molecular response evaluation was based on quantitative *BCR::ABL1* transcript levels obtained by reverse transcription polymerase chain reaction (RT-qPCR) and expressed as the percentage ratio of *BCR::ABL1* to ABL1 transcripts (*BCR::ABL1/ABL1* × 100), reported on the International Scale using a standardized conversion factor.

*BCR::ABL1* values were also expressed in logarithmic reductions, where transcript levels of 1%, 0.1%, 0.01%, 0.0032%, and 0.001% correspond to 2-, 3-, 4-, 4.5-, and 5-log reductions, respectively [16, 21]. Molecular assessments were grouped according to predefined time intervals: 6 months (5–8 months), 12 months (11–14 months), 18 months (17–20 months), and 24 months (23–26 months) for patients treated with imatinib; and 6, 12, and 18 months for those treated with dasatinib or nilotinib.

Molecular response categories were defined according to European LeukemiaNet recommendations applicable during the study period and classified descriptively as favorable (optimal), warning, or unfavorable (failure) at each evaluated timepoint. Major molecular response (MMR) was defined as *BCR::ABL1* ≤ 0.1% on the International Scale, and deep molecular response (DMR) as *BCR::ABL1* ≤ 0.01% (MR4 or deeper). Sustained deep molecular response (sDMR) was defined as maintenance of DMR for at least two consecutive years in patients with a minimum follow-up of four years [16, 21].

### Descriptive and statistical analysis

Statistical analyses were descriptive in nature. Data were tabulated using Microsoft Excel^®^ spreadsheets and analyzed with GraphPad Prism software (version 8.0). Categorical variables were summarized as absolute numbers (*n*) and percentages (%), while continuous variables were expressed as mean ± standard deviation and presented in tables and figures.

The annual incidence profile was derived from the number of newly diagnosed cases between 2011 and 2020. Clinical variables, including disease phase and Sokal risk score, as well as laboratory parameters such as *BCR::ABL1* transcript subtype and molecular response categories, were analyzed according to their respective classifications.

## Results

### Annual incidence, cohort profile, and treatment flowchart in patients with CML

**Fig. 1:**
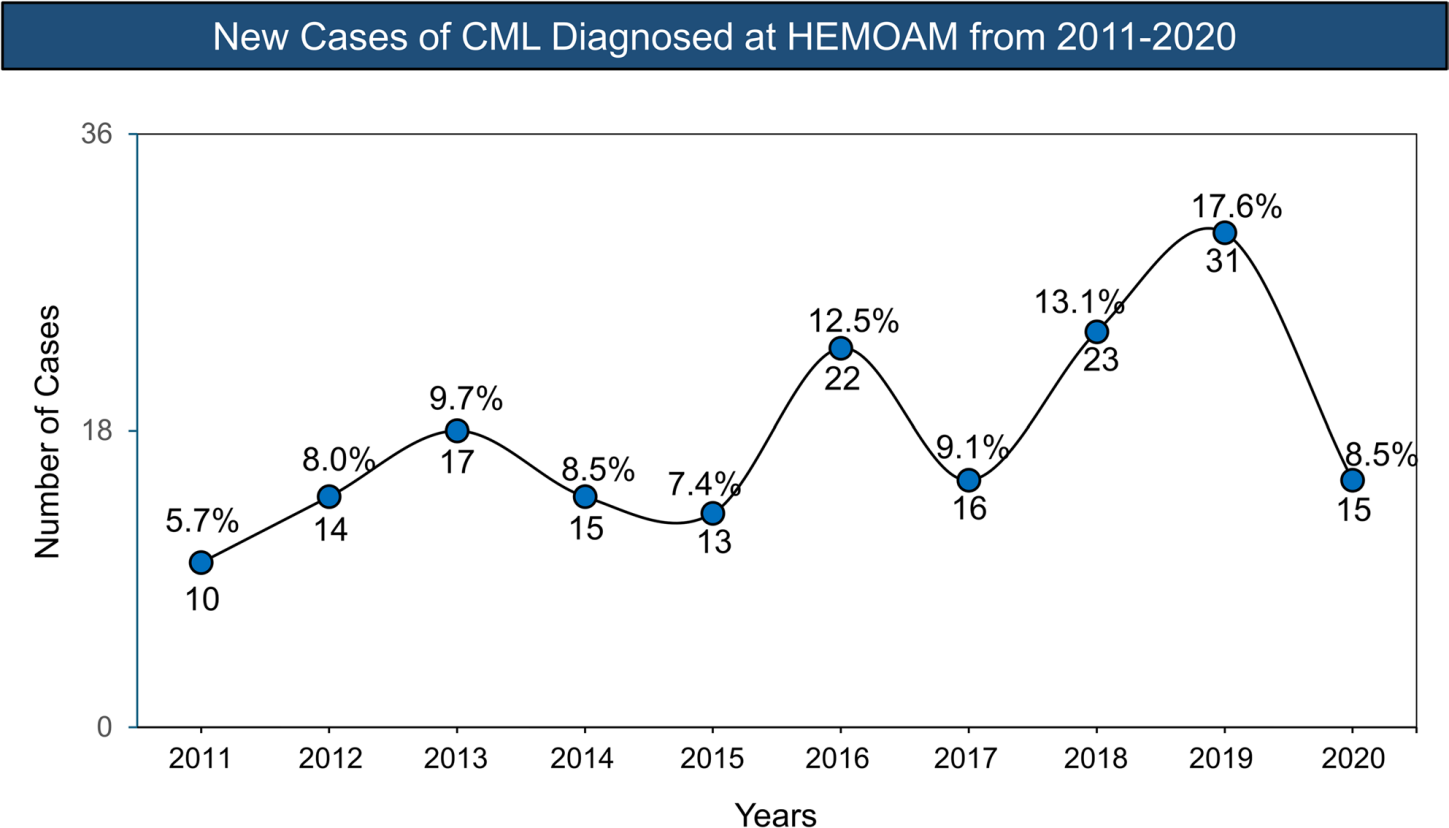
Annual incidence of CML cases at HEMOAM from 2011 to 2020. Source: HEMOAM (2022)

This retrospective study included 176 patients diagnosed with chronic myeloid leukemia (CML) at HEMOAM between January 2011 and December 2020. The annual number of new diagnoses varied over time, ranging from 10 cases in 2011 to a peak of 31 cases in 2019 (Fig. 1). Among these patients, 122 diagnosed in the chronic phase were included in the longitudinal clinical and molecular analyses, while 54 were excluded due to absence of molecular monitoring data or loss to follow-up.

**Fig. 2:**
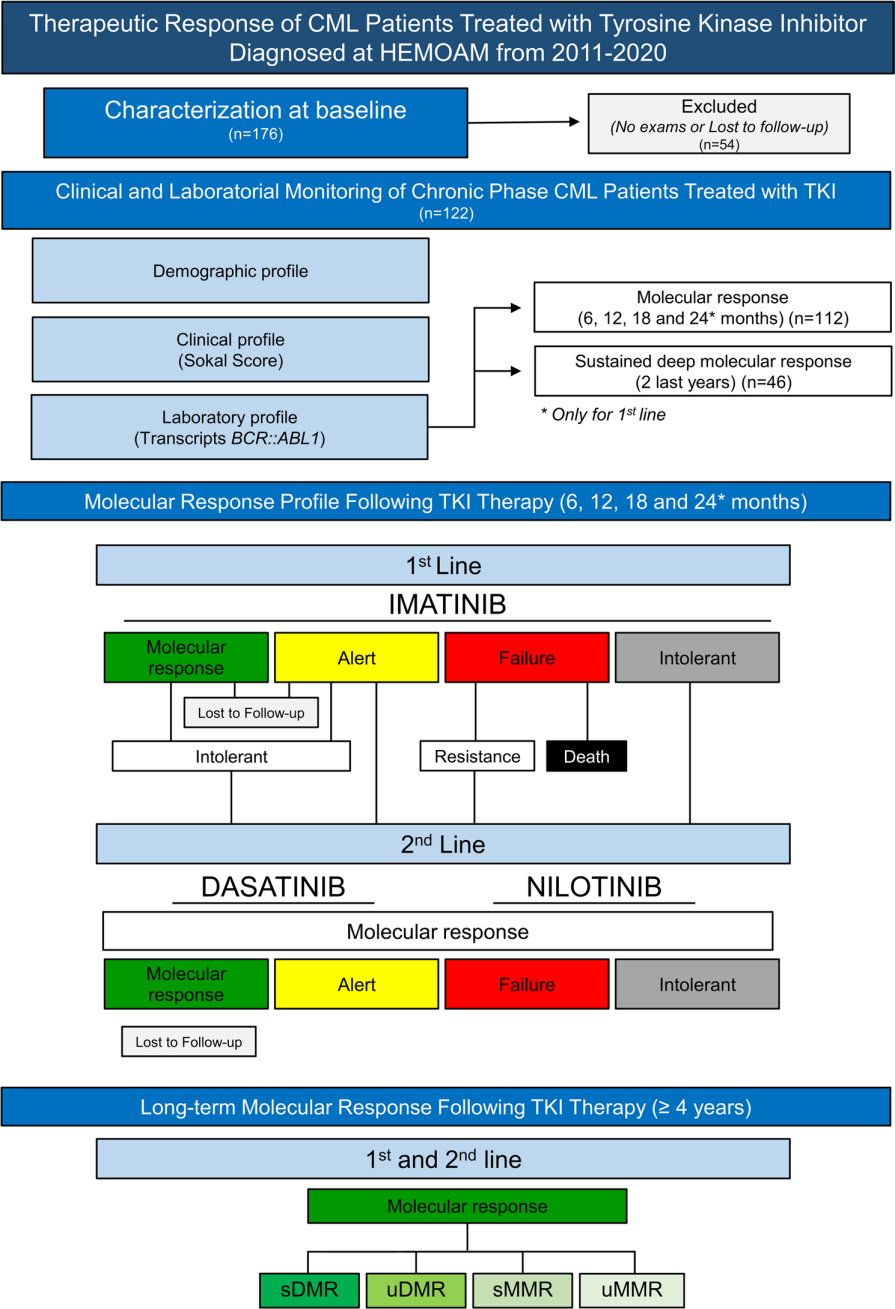
Therapeutic response profile of patients diagnosed with chronic myeloid leukemia (CML) and treated with tyrosine kinase inhibitors (TKIs) at HEMOAM between 2011 and 2020. The flowchart summarizes baseline demographic, clinical, and laboratory characteristics (*n* = 176), distribution of *BCR::ABL1* transcript types, and molecular response at 6, 12, 18, and 24 months (*n* = 122). Outcomes are shown according to first-line imatinib and second-line therapy with dasatinib or nilotinib, including categories of molecular response, warning, failure, intolerance, resistance, and loss to follow-up. Sustained deep molecular response (sDMR) was evaluated in patients with ≥ 4 years of follow-up (*n* = 46). Abbreviations - sDMR: sustained deep molecular response; uDMR: unfavorable deep molecular response; sMMR: sustained major molecular response; uMMR: unfavorable major molecular response

Most patients initiated treatment with imatinib as first-line therapy (*n* = 110). During follow-up, patients were stratified according to treatment line, molecular response category, and clinical outcome, including transition to second-line therapy and sustained molecular responses (Fig. 2). Molecular assessments closest to predefined timepoints, 6 (5–8), 12 (11–14), 18 (17–20), and 24 (23–26) months, were included. Thirty-three patients were switched to second-generation TKIs (dasatinib or nilotinib) due to treatment failure, intolerance, or persistent warning response. Final outcomes were defined at the last available follow-up, including evaluation of sustained molecular responses in patients with long-term follow-up.

### Demographic, clinical and laboratory characterization of the study population

The study population consisted predominantly of adults, with a mean age at diagnosis of 49.6 ± 13.7 years (range, 22–79 years). There was a slight male predominance, with 66 males (54.1%) and 56 females (45.9%).

Regarding geographic origin, most patients resided in Manaus, the capital of Amazonas (*n* = 92; 75.4%), while 26 patients (21.3%) originated from municipalities in the interior of the state and four (3.3%) from other Brazilian states (Table 1). All patients included in the analysis were diagnosed in the chronic phase of CML.

**Table 1:** Demographic, clinical, and laboratory characteristics of patients diagnosed with Chronic Myeloid Leukemia (CML) at HEMOAM from Jan 1st, 2011 to Dec 31st, 2020

Parameters	CML Patients (*n* = 122)
Gender	
Female (%)	56 (45.9%)
Male (%)	66 (54.1%)
Age, years (mean ± SD)	49.6 ± 13.7
Origin	
State Capital - Manaus (%)	92 (75.4%)
Interior of Amazonas* (%)	26 (21.3%)
Other States** (%)	4 (3.3%)
Disease phase	
Chronic (%)	122 (100%)
Sokal score	
LR (Low risk): < 0.8	6 (4.9%)
IR (Intermediate risk): 0.8–1.2	14 (11.5%)
HR (High risk): > 1.2	50 (41.0%)
Not characterized	52 (42.6%)
Transcript type	
b3a2	62 (50.8%)
b2a2	45 (36.9%)
b2a2/b3a2	1 (0.8%)
Not performed	14 (11.5%)

* Apuí, Barreirinha, Beruri, Borba, Coari, Codajás, Guajará, Iranduba, Manacapuru, Manaquiri, Parintins, Presidente Figueiredo, Rio Preto da Eva, São Gabriel da Cachoeira, Tabatinga

** Pará (PA) and Roraima (RR)

Sokal risk score was available for 70 patients (57.4%), among whom 41.0% were classified as high-risk, 11.5% as intermediate-risk, and 4.9% as low-risk. In 52 patients (42.6%), Sokal score could not be calculated due to missing baseline data, particularly spleen size at diagnosis, which limits the interpretability of risk stratification in this cohort.

*BCR::ABL1* transcript type was available for 108 patients (88.5%). The most frequent transcript was b3a2 (e14a2), identified in 62 patients (50.8%), followed by b2a2 (e13a2) in 45 patients (36.9%). One patient (0.8%) presented both transcripts. Transcript data were unavailable for 14 patients (11.5%) because molecular testing was performed outside HEMOAM (Table 1).

### Molecular response characterization of CML patients treated with first- and second-line tyrosine kinase inhibitors

Among the 122 patients included in the analysis, all had at least one molecular assessment during follow-up. However, the number of patients evaluated at each predefined timepoint varied substantially, primarily due to unavailability of molecular testing at specific intervals (Table 2).

**Table 2:**
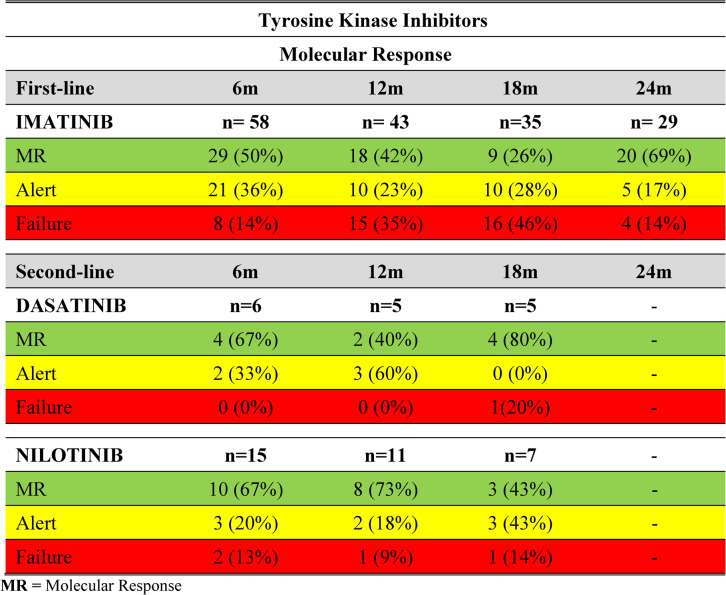
Molecular response profile at 6, 12, 18, and 24 months of CML patients at HEMOAM treated with 1st and 2nd line Tyrosine Kinase Inhibitors according to the European Leukemia Net (ELN) guidelines

Importantly, patients evaluated at different timepoints do not represent a fixed longitudinal cohort. Higher molecular response rates observed at later assessments reflect a selected subgroup of patients with available molecular monitoring, many of whom had achieved response at earlier timepoints. Therefore, variations in response rates across timepoints should be interpreted descriptively and not as true longitudinal improvement or decline at the individual patient level.

**Fig. 3:**
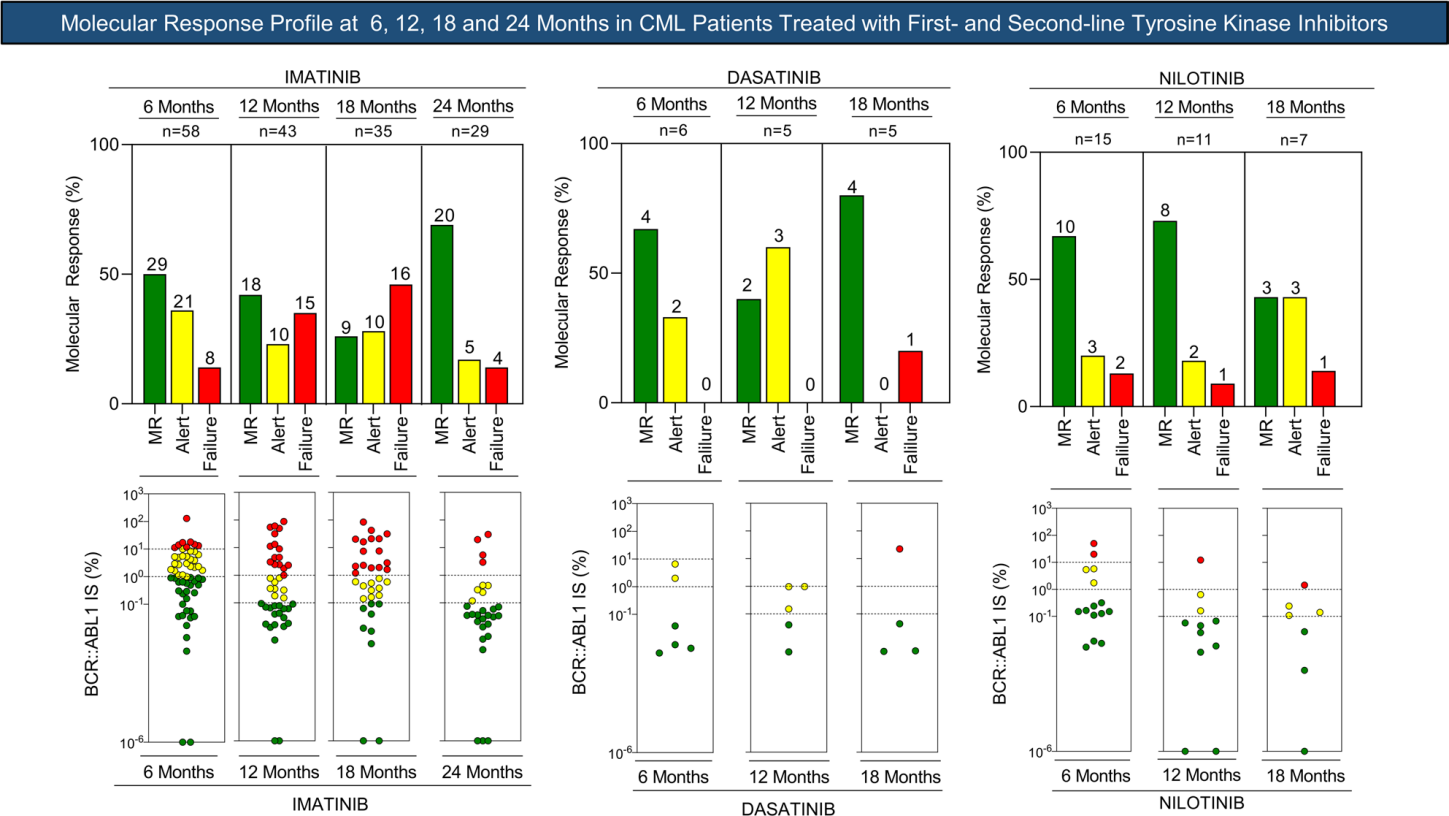
Molecular response profile at 6, 12, 18, and 24 months in CML patients undergoing first- and second-line tyrosine kinase inhibitor therapy, according to European LeukemiaNet (ELN) guidelines

**Fig. 4:**
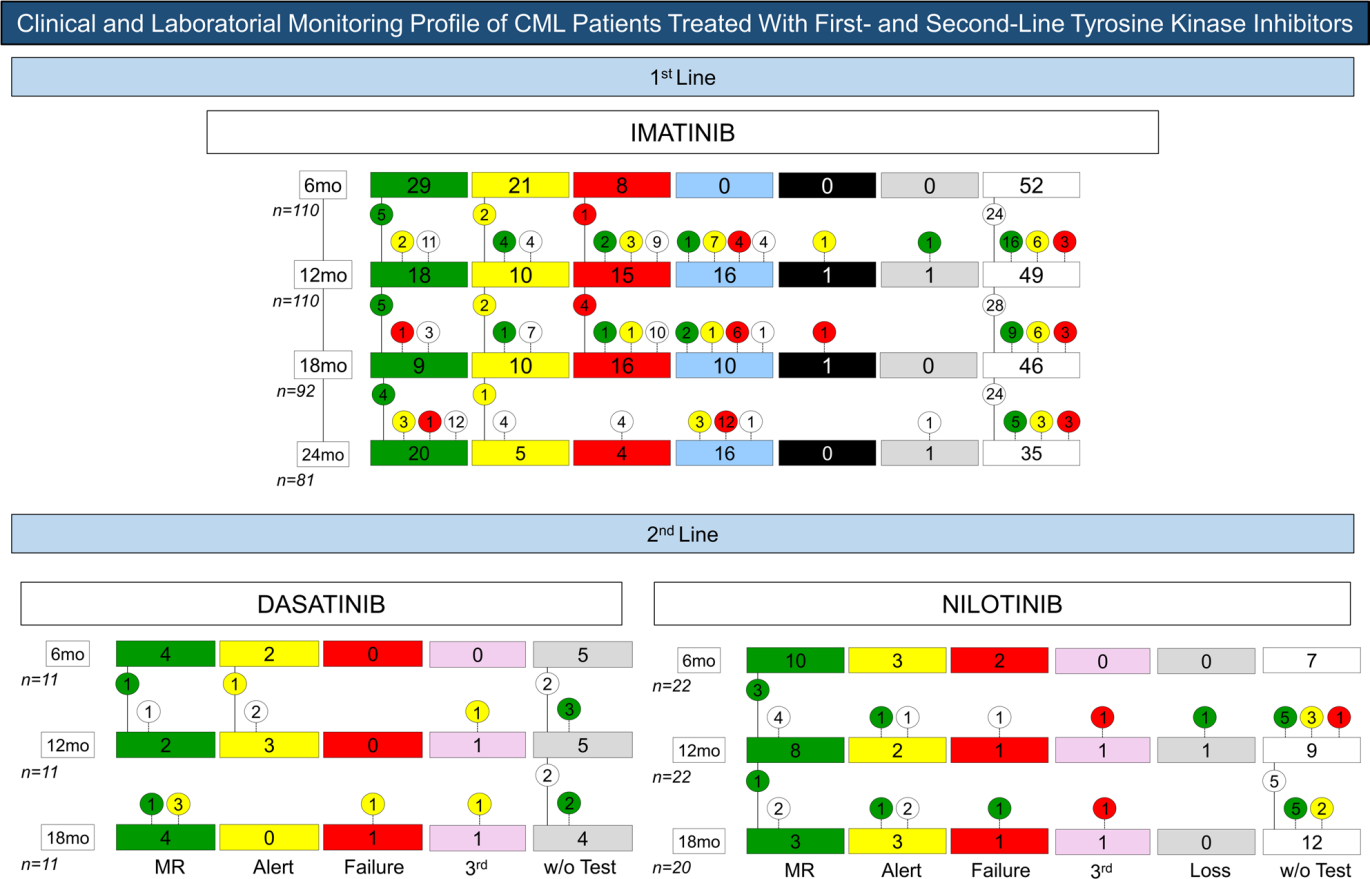
Follow-up of the clinical and laboratory profile of patients receiving first- and second-line tyrosine kinase inhibitor therapy, classified according to molecular response, including categories of warning, failure, transition to second-line therapy, death, loss to follow-up, or absence of tests during the evaluated period (w/o test)

Among patients who initiated imatinib as first-line therapy (*n* = 110), the number of evaluable patients varied across timepoints due to missing molecular assessments. Detailed distributions of response categories (favorable, warning, and unfavorable) at each timepoint are presented in Table 2 and Figs. 3 and 4.

Overall, patients classified as having favorable molecular response at early timepoints were more likely to maintain response during follow-up, whereas those categorized as warning or unfavorable more frequently required transition to second-line therapy. Detailed individual response trajectories, treatment switches, and clinical outcomes are summarized in Figs. 3 and 4.

### Molecular response characterization of CML patients treated with second-line tyrosine kinase inhibitors

A total of 33 patients were treated with second-generation TKIs, including 11 receiving dasatinib and 22 receiving nilotinib. Molecular response was evaluated at 6, 12, and 18 months when data were available.

Among patients treated with dasatinib, molecular response was observed in 67% at 6 months (4/6) and 80% at 18 months (4/5). At final follow-up, molecular response was documented in eight of 11 patients (72.7%), while two patients remained in warning and one experienced treatment failure. Some patients were not assessed at intermediate timepoints due to missing molecular data.

In the nilotinib-treated group, molecular response rates were 67% at 6 months (10/15), 73% at 12 months (8/11), and 43% at 18 months (3/7). At the final assessment, nine of 22 patients (41%) were classified as having molecular response, while eight (36.4%) were in warning and four (18.2%) in failure. Missing molecular assessments were frequent at later timepoints in this group.

All comparisons between second-generation TKIs are descriptive and should be interpreted cautiously due to small sample sizes, heterogeneous follow-up, and selection bias.

### Sustained deep molecular response and longitudinal flow throughout the study

**Fig. 5:**
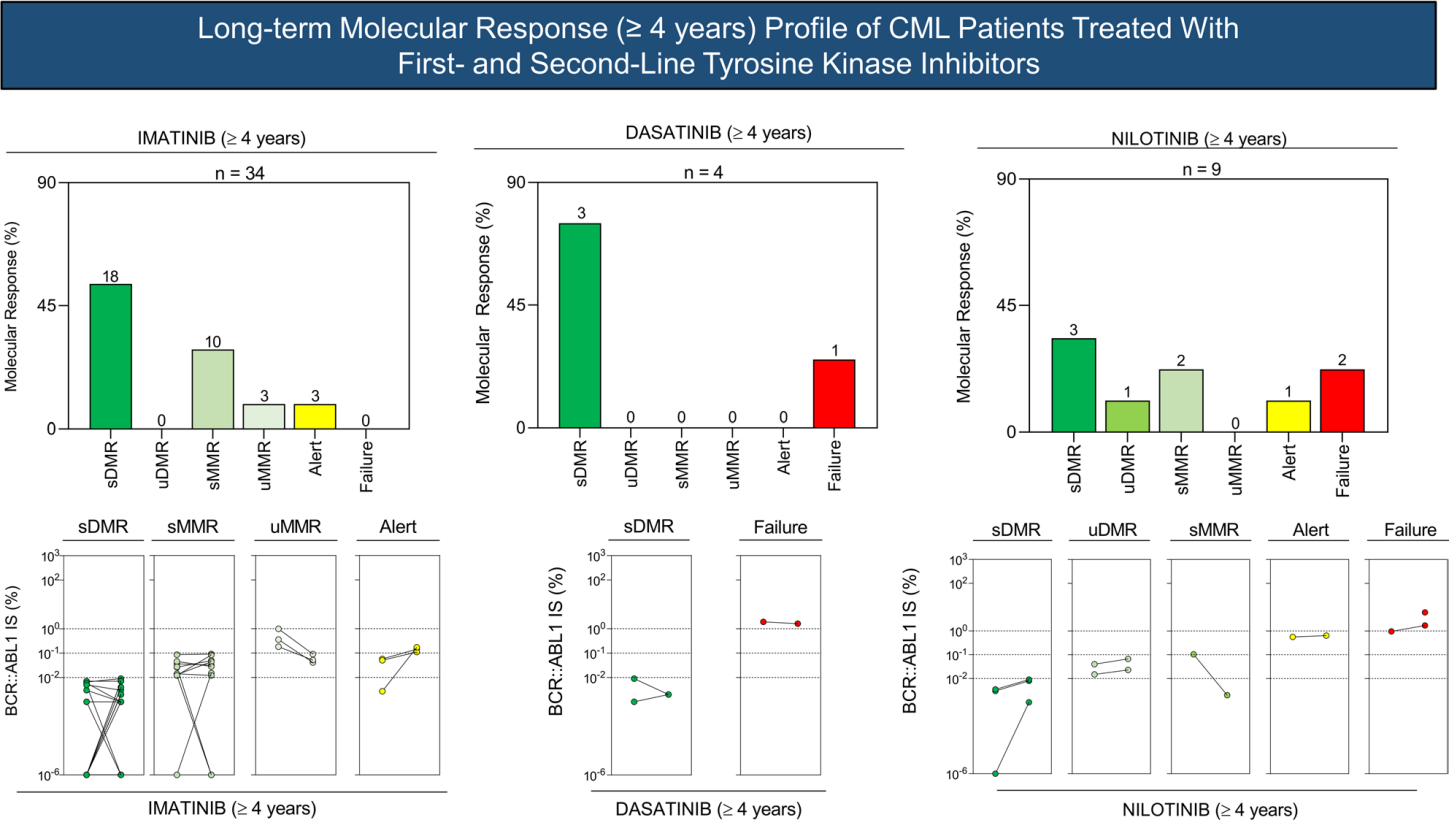
Profile of Deep molecular Response (DMR) in CML patients treated with first- and second-line tyrosine kinase inhibitors (TKIs) for more than 4 years, according to European LeukemiaNet (ELN) guidelines

**Fig. 6:**
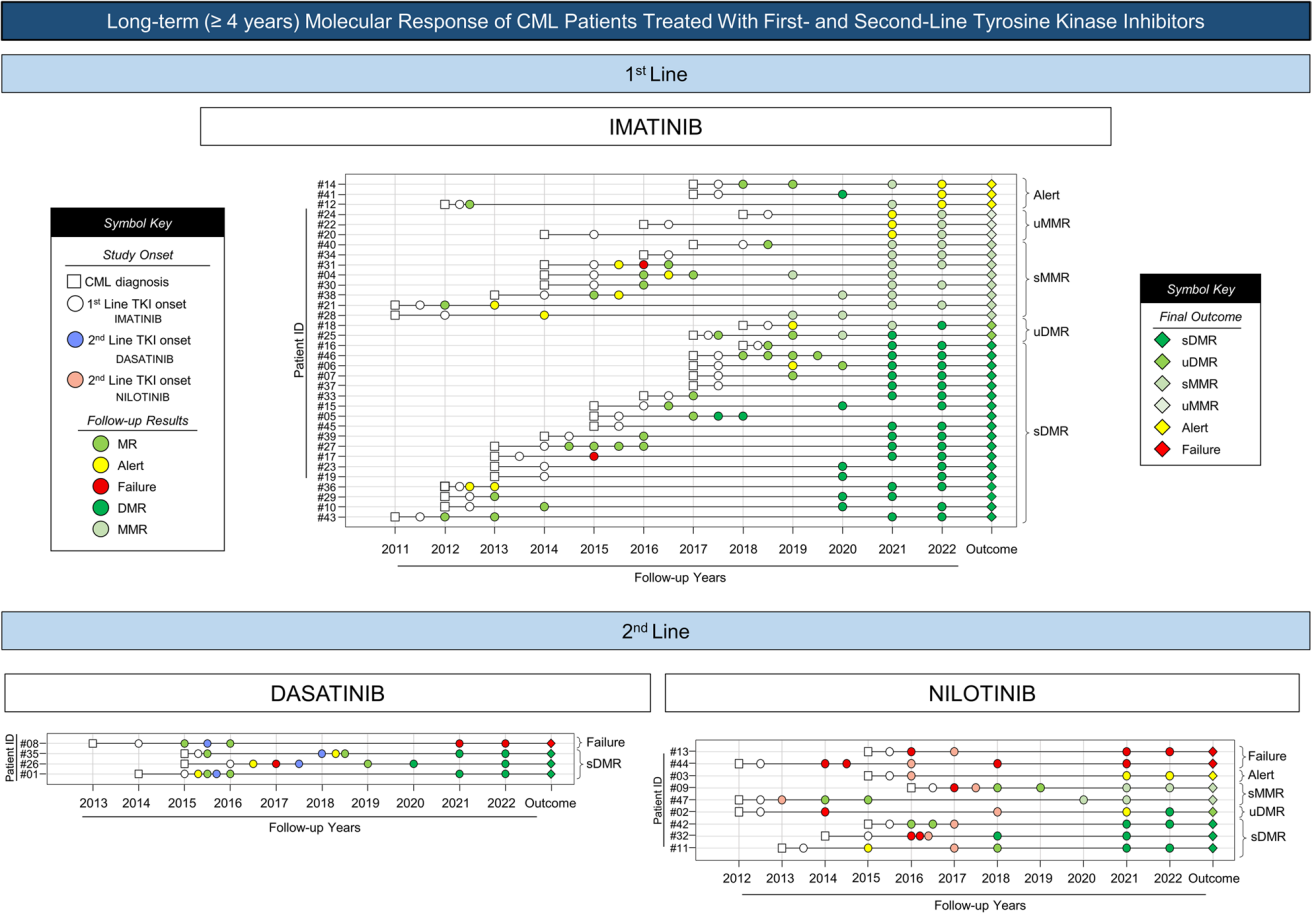
Long-term follow-up of 34 CML patients treated with tyrosine kinase inhibitors for ≥4 years, depicting the follow-up design throughout the study period and their final outcomes

Sustained deep molecular response (sDMR) was evaluated in patients with a minimum follow-up of four years who achieved deep molecular response (DMR), defined as *BCR::ABL1* ≤ 0.01% (MR4 or deeper), maintained for at least two consecutive years. Patients were classified as having sustained or unsustained DMR, sustained or unsustained major molecular response (MMR), warning, or failure (Table 3 Figs. 5 and 6).

**Table 3:**
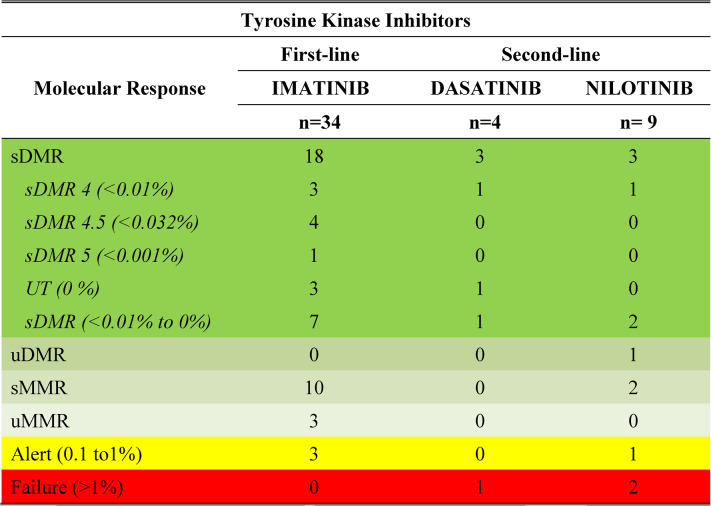
Deep Molecular response (DMR) profile of CML patients treated with 1st and 2nd line Tyrosine Kinase Inhibitors for more than 4 years and sustained for at least 2 years, according to the European Leukemia Net (ELN) guidelines

Among patients treated with imatinib, a predominance of sustained DMR and sustained MMR was observed, although fluctuations in molecular response occurred in some individuals. In the dasatinib-treated group, most patients achieved sustained DMR, with one case of treatment failure. In contrast, patients treated with nilotinib as second-line therapy showed a more heterogeneous response profile, including sustained DMR, unsustained responses, warning, and failure categories.

Longitudinal molecular response patterns for patients with long-term follow-up are summarized in Fig. 6.

## Discussion

This study provides a real-world overview of Philadelphia chromosome–positive chronic myeloid leukemia (CML) in the state of Amazonas, Brazil, describing incidence trends, demographic and clinical characteristics, and longitudinal molecular monitoring in patients treated at HEMOAM between 2011 and 2020. Beyond characterizing outcomes, our findings highlight how geographic distance, referral pathways, and intermittent access to standardized molecular testing shape CML management in routine practice in the Amazon region.

A total of 176 new cases were identified during the study period, with annual diagnoses ranging from 10 to 22 cases up to 2017, increasing to 31 cases in 2019, and decreasing to 15 cases in 2020. This reduction in 2020 is plausibly related to the COVID-19 pandemic, which disrupted healthcare access and reduced diagnostic activity in multiple settings. In Amazonas, the need for referral through the state regulation system (SISREG) represents an additional barrier, particularly for remote municipalities where shortages of specialized physicians and long river travel to Manaus can delay diagnosis and continuity of care [22].

To mitigate barriers in the region, HEMOAM implemented technical training initiatives between 2017 and 2020 focused on blood count interpretation and cell morphology for laboratory professionals from multiple municipalities. In parallel, institutional investments were made to expand local diagnostic capacity, including the progressive incorporation of molecular testing infrastructure.

The demographic profile of our cohort was consistent with prior Brazilian studies, with a mean age at diagnosis of 49.6 years and slight male predominance [23–25]. The lower mean age compared with some international cohorts (typically 55–60 years) may reflect differences in population structure, referral patterns, and healthcare access [16, 26, 27]. Most patients originated from Manaus; however, a substantial proportion came from municipalities in the interior of Amazonas and neighboring states, reinforcing HEMOAM’s role as a regional reference center and illustrating the logistical complexity of care in this setting [28, 29].

All patients included in the longitudinal analyses were diagnosed in the chronic phase of CML, which is expected given the typically indolent course of the disease and the widespread availability of diagnostic testing in the TKI era [30, 31]. Sokal score could be calculated in 70 patients, among whom most were classified as high-risk, followed by intermediate- and low-risk categories, consistent with previous reports describing heterogeneous risk distribution at diagnosis [32, 33]. However, more than 40% of patients lacked sufficient baseline information, particularly spleen size at diagnosis, which limited the applicability and interpretability of prognostic stratification in this cohort. Although the European LeukemiaNet (ELN) 2020 guidelines recommend the ELTS (EUTOS Long-Term Survival) score as the preferred tool for predicting CML-related mortality, ELTS could not be calculated due to incomplete baseline variables, reducing comparability with contemporary cohorts and reflecting real-world documentation challenges [16].

Regarding *BCR::ABL1* transcript subtypes, e14a2 (b3a2) predominated in our population, a pattern consistent with national and international cohorts [34]. Transcript subtype has been explored as a potential prognostic modifier, with some studies suggesting associations with early molecular response and treatment-free remission rates. In Brazil, higher early molecular response rates among patients carrying the e14a2 transcript have been reported, although without significant differences in overall survival, progression-free survival, or event-free survival [35]. International studies similarly suggest improved molecular response kinetics and longer event-free survival among b3a2-positive patients treated with imatinib or nilotinib, although these findings have not been uniformly observed across all cohorts [19, 36, 37].

The management of CML is centered on tyrosine kinase inhibitors, whose efficacy is monitored through quantitative RT-PCR assessment of *BCR::ABL1* transcripts, allowing longitudinal evaluation of treatment response and timely therapeutic adjustments [14, 16]. In our cohort, molecular response distributions varied across predefined timepoints; however, these variations should not be interpreted as evidence of biological resistance or recovery. Response assessments involved partially overlapping patient subsets and were influenced by test availability, adherence, and continuity of follow-up [16, 26]. Higher response rates observed at later timepoints likely reflect selection of patients with prior response and available molecular monitoring, as also described in other real-world studies [17, 38].

When contextualized against landmark clinical trials and large institutional series, molecular response proportions in our imatinib-treated patients at 12 and 24 months were within the range reported in the literature. In the IRIS trial, approximately 39% of patients achieved major molecular response (MMR) at 12 months and 69% at 24 months, values comparable to those observed in our cohort [39]. Higher response rates reported in highly controlled settings, such as the MD Anderson Cancer Center experience, may reflect stricter adherence monitoring, systematic follow-up, and uninterrupted access to standardized molecular testing, factors known to influence observed outcomes [17, 38, 40].

Evaluation of ELN milestones further contextualizes these findings. According to the 2013 ELN recommendations, expected MMR rates are ≥ 36% at 6 months, ≥ 58% at 12 months, and ≥ 78% at 18 months. In our cohort, outcomes at 6 and 24 months approached these benchmarks, whereas intermediate timepoints showed lower performance, likely reflecting challenges common to public healthcare systems, including irregular molecular monitoring, treatment interruptions, and adherence difficulties. These observations reinforce the importance of systematic follow-up and early molecular response as predictors of long-term outcomes [41].

National real-world data provide additional perspective. In a multicenter Brazilian cohort, Vieira-Mion et al. reported MMR rates of 44% at 12 months and 63% at 24 months among patients treated with imatinib in the public health system [42]. Although molecular response at intermediate timepoints in our study was lower, the 24-month response rate was comparable to national benchmarks. Temporal fluctuations in response may be influenced by delayed treatment initiation, inconsistent adherence, laboratory variability, and limited access to molecular diagnostics, particularly in resource-constrained settings.

In routine clinical practice, treatment interruptions were sometimes associated with asymptomatic disease, leading some patients to discontinue therapy autonomously, with relapse detected only upon laboratory reassessment. This underscores the importance of evaluating adherence before modifying treatment strategies, in line with international recommendations, to avoid unnecessary treatment switches and additional toxicity [4, 16]. Achieving molecular response after a warning or failure category may reflect improved adherence, whereas confirmed failure at any timepoint is associated with inferior progression-free survival and increased risk of disease transformation [43].

In our cohort, most transitions to second-line therapy occurred due to confirmed treatment failure, followed by persistent warning response and intolerance. Progression to blast crisis or death was infrequent, emphasizing the benefit of early detection of suboptimal response and adherence monitoring. Although some patients classified as warning eventually achieved molecular response, delayed responses have been associated with lower probability of achieving deep molecular remission and higher risk of subsequent loss of response [44].

Molecular responses observed with dasatinib as second-line therapy followed trends described in real-world studies. In a French cohort of patients resistant or intolerant to imatinib, approximately 49% achieved MMR at 12 months, comparable to results from the PEARL study [45]. These findings suggest consistent performance of dasatinib in rescue settings, despite variability in patient characteristics and monitoring practices.

In our analysis, the 6-month molecular response rate observed with dasatinib was within the range reported in pivotal and real-world studies [46]. Progressive deepening of response over time has also been described in longer-term follow-up studies, including DASISION [16]. At later timepoints, treatment failure rates in our cohort were within the range reported in international real-world series, supporting the external validity of these observations [37, 47].

Among patients treated with nilotinib as second-line therapy, early molecular response rates were consistent with those reported in clinical trials such as GIMEMA and ENESTnd, as well as in observational studies [47, 48]. The decline observed at later timepoints may reflect treatment interruptions, toxicity, acquired resistance, or suboptimal adherence, factors that are particularly relevant in real-world settings [49]. A substantial proportion of patients remained classified as warning according to ELN 2020 criteria, highlighting the need for close monitoring and timely intervention [16]. Overall, second-generation TKIs remain effective therapeutic options for patients intolerant or resistant to imatinib. Differences in toxicity profiles, dosing schedules, and drug–drug interactions necessitate individualized treatment decisions and continuous clinical surveillance rather than uniform sequencing strategies [16, 37, 50, 51]. Sustained deep molecular response (sDMR) represents a clinically relevant endpoint in CML, as it enables treatment discontinuation and treatment-free remission in selected patients. In our cohort, sDMR was observed in both dasatinib- and nilotinib-treated patients, although the small number of evaluable cases limits direct comparison. Reported rates are consistent with findings from trials such as ENESTcmr and DASFREE, which demonstrated durable deep responses in a proportion of patients receiving second-line TKIs [52, 53]. Evidence from discontinuation trials, including EURO-SKI and STIM, indicates that approximately 40–65% of patients achieving sustained deep molecular response can maintain treatment-free remission, underscoring the importance of rigorous and continuous molecular monitoring [54–56]. In this context, our findings highlight the challenges of implementing treatment-free remission strategies in regions with intermittent access to standardized molecular testing.

Limited availability of *BCR::ABL1* testing at HEMOAM until 2020 represented a major barrier to optimal molecular monitoring. Prior to local implementation, testing depended on external sponsorship and was not consistently aligned with clinical visits, contributing to incomplete longitudinal assessment.

This study has important limitations inherent to its retrospective, real-world design. High proportions of missing molecular assessments reflect structural and logistical constraints rather than random data loss. Patients with available molecular testing at later timepoints likely represent a selected subgroup with better adherence or access to care, and response rates may therefore overestimate population-level outcomes. In addition, incomplete baseline data limited risk stratification using Sokal or ELTS scores. Taken together, these findings underscore the complexity of interpreting longitudinal molecular response patterns in real-world settings and the need for adapted strategies to improve access to standardized monitoring in geographically remote regions.

## Conclusion

This decade-long real-world study (2011–2020) conducted at the Fundação Hospitalar de Hematologia e Hemoterapia do Amazonas (HEMOAM) provides an overview of chronic myeloid leukemia (CML) management in the state of Amazonas, highlighting demographic characteristics, treatment patterns, and longitudinal molecular monitoring in routine clinical practice.

Our findings illustrate how molecular monitoring is applied in a geographically and logistically challenging setting and show that meaningful molecular responses can be achieved in both first- and second-line treatment contexts. Observed response patterns underscore the importance of sustained access to standardized *BCR::ABL1* testing and careful interpretation of molecular milestones in real-world populations with incomplete follow-up.

Despite substantial geographic barriers affecting patient monitoring and continuity of care, recent institutional investments in local molecular diagnostics represent an important step toward improving disease monitoring and therapeutic decision-making. Overall, this study emphasizes the critical role of continuous molecular monitoring to support individualized treatment strategies and provides a foundation for future prospective initiatives, including structured evaluations of treatment-free remission (TFR) feasibility in patients with CML from the Amazon region. 

## Data Availability

Due to ethical and privacy restrictions, the data supporting the findings of this study cannot be made publicly available. However, anonymized data may be made available from the corresponding author upon reasonable request and under a data sharing agreement.

## Abbreviations

CML: Chronic myeloid leukemia
TKI: Tyrosine kinase inhibitor
BCR: ABL1:Breakpoint cluster region-Abelson fusion gene
MR: Molecular response
MMR: Major molecular response
DMR: Deep molecular response
sDMR: Sustained deep molecular response
ELN: European LeukemiaNet
